# Influence of Age on Hyperoxia-Induced Cardiac Pathophysiology in Type 1 Diabetes Mellitus (T1DM) Mouse Model

**DOI:** 10.3390/cells12111457

**Published:** 2023-05-24

**Authors:** Faizan Saleem, Noah Mansour, Riddhi Vichare, Yashwant Ayalasomayajula, Jenna Yassine, Anagha Hesaraghatta, Siva Kumar Panguluri

**Affiliations:** 1 Department of Pharmaceutical Sciences, College of Pharmacy, University of South Florida, 12901 Bruce B. Downs Blvd., Tampa, FL 33612, USA; faizansaleem@usf.edu (F.S.); hamansour@usf.edu (N.M.); vicharer@duq.edu (R.V.); yashwant@usf.edu (Y.A.); jennayassine@usf.edu (J.Y.); anaghah@usf.edu (A.H.); 2 Cell Biology, Microbiology and Molecular Biology, College of Arts and Sciences, University of South Florida, 12901 Bruce B. Downs Blvd., Tampa, FL 33612, USA

**Keywords:** type 1 diabetes, hyperoxia, potassium channels, cardiovascular disease (CVD), aging, mechanical ventilation

## Abstract

Mechanical ventilation often results in hyperoxia, a condition characterized by excess SpO_2_ levels (>96%). Hyperoxia results in changes in the physiological parameters, severe cardiac remodeling, arrhythmia development, and alteration of cardiac ion channels, all of which can point toward a gradual increase in the risk of developing cardiovascular disease (CVD). This study extends the analysis of our prior work in young Akita mice, which demonstrated that exposure to hyperoxia worsens cardiac outcomes in a type 1 diabetic murine model as compared to wild-type (WT) mice. Age is an independent risk factor, and when present with a major comorbidity, such as type 1 diabetes (T1D), it can further exacerbate cardiac outcomes. Thus, this research subjected aged T1D Akita mice to clinical hyperoxia and analyzed the cardiac outcomes. Overall, aged Akita mice (60 to 68 weeks) had preexisting cardiac challenges compared to young Akita mice. Aged mice were overweight, had an increased cardiac cross-sectional area, and showed prolonged QTc and JT intervals, which are proposed as major risk factors for CVD like intraventricular arrhythmias. Additionally, exposure to hyperoxia resulted in severe cardiac remodeling and a decrease in Kv 4.2 and KChIP2 cardiac potassium channels in these rodents. Based on sex-specific differences, aged male Akita mice had a higher risk of poor cardiac outcomes than aged females. Aged male Akita mice had prolonged RR, QTc, and JT intervals even at baseline normoxic exposure. Moreover, they were not protected against hyperoxic stress through adaptive cardiac hypertrophy, which, at least to some extent, is due to reduced cardiac androgen receptors. This study in aged Akita mice aims to draw attention to the clinically important yet understudied subject of the effect of hyperoxia on cardiac parameters in the presence of preexisting comorbidities. The findings would help revise the provision of care for older T1D patients admitted to ICUs.

## 1. Introduction

Oxygen supplementation through mechanical ventilation (MV) to combat hypoxia is an integral part of resuscitation programs in intensive care units (ICUs) [1]. Oxygen therapy is frequently administered to critically ill patients who have experienced episodes of cardiac arrest, sepsis, respiratory failure, traumatic brain injury (TBI), or any major surgeries [2]. Recently, oxygen therapy was a frontline treatment for hospitalized patients with hypoxemic respiratory failure due to severe acute respiratory syndrome coronavirus 2 (SARS-CoV-2) pneumonia [1]. Despite the benefits, several cautionary reports cite that 16–50% of mechanically ventilated patients experience hyperoxia because of liberal oxygen supplementation [3]. These reports also draw attention to poorly regulated clinical guidelines for oxygen delivery and a lack of cutoff limits for the target oxygen saturation (SpO_2_) or partial oxygen pressure (PaO_2_) [4]. Hyperoxia occurs when SpO_2_ is greater than 96% or patients are exposed to supranormal values of PaO_2_ (>150 mm Hg) [5]. Li et al. [6] reported that one to five hours of hyperoxia precipitates ventilator-induced lung injury by upregulating the production of the cytokine macrophage inflammatory protein-2 (MIP-2) and activation of mitogen-activated protein kinase pathways in a rodent model. Similarly, Sinclair et al. [7] reported increased lung injury scores, histopathological changes, and increased production of chemokines and neutrophil counts in white rabbits. Other consequences of hyperoxia include the generation of reactive oxygen species and increased alveolar permeability, which causes an influx of inflammatory cells [8]. Inevitably, meta-analyses of clinical trials have reported poor patient outcomes and a significant association between increased risk of mortality and hyperoxia [9].

Although direct lung toxicity because of hyperoxia is well documented [7], the Air Versus Oxygen in Myocardial Infarction (AVOID) trial identified evidence suggesting an increased risk of developing myocardial injury after oxygen supplementation [10]. Moreover, exposure to hyperoxia during MV is reported to decrease coronary blood flow and myocardial oxygen consumption [11]. Previously, we have reported severe cardiac remodeling, decreased cardiac output, dysregulation of cardiac potassium channels, and an increased cardiac troponin marker as effects of long-term exposure to hyperoxia in young (8 to 9 weeks) small rodents [12,13]. Nevertheless, there is still only a narrow insight into the development of cardiac functional and structural changes in patients with preexisting comorbidities who receive mechanical ventilation. In another rodent study [14], our group showed that advanced age, as a preexisting condition, is not only an independent risk factor for cardiac remodeling, but that the presence of hyperoxia further exacerbates physiological and functional cardiac outcomes, thus increasing the risk of cardiovascular disease (CVD). However, a major cohort of patients admitted to the ICU consists of aged individuals with comorbidities like diabetes [15]. Diabetes is a major health complication in the geriatric population, with an estimated 50–80% of older patients being either prediabetic or diabetic [16]. As a result, older patients with coexisting diabetes are frequently admitted to the ICU for managing complications, and therefore have higher odds of being exposed to hyperoxic episodes.

Diabetes is broadly classified into type 1 (insulin-dependent diabetes mellitus, TIDM) and type 2 (non-insulin dependent diabetes mellitus, T2DM) [17]. Rodgers et al. [18] demonstrated that hyperoxic T2DM mice (B6 dB mice, 8 to 10 weeks) were more susceptible to developing arrhythmias than the hyperoxic heterozygous controls. Although the majority of patients admitted to ICUs have T2DM, patients with T1DM make up anywhere between 9.3% and 25% of the total ICU patient population [19]. Therefore, it is imperative to study the effect of oxygen supplementation on cardiac parameters in T1DM patients. T1DM is not only a major risk factor for CVD, but is also a commonly reported comorbidity in MV patients [20]. Bojkovic et al. [21] reported that exposure to hyperoxia resulted in the worst cardiac outcomes in young T1DM mice (Akita, 10 weeks) as compared to the wild-type (WT) controls. Moreover, young male Akita mice had a higher sex-dependent risk of developing CVD post-hyperoxia than young female Akita mice. The prevalence of CVD increases by 6% in young T1DM patients (age 15–29), but a dramatic 25% increase is observed in aged T1DM patients (age 45–59) [22]. Thus, this research primarily focuses on determining how long-term hyperoxia affects cardiac outcomes in an aged (60 to 68 weeks) T1DM murine model. We investigated the impact of aging alone in T1DM mice by comparing data from our previously published work in young T1DM mice [21]. This study also draws attention to the sex disparities between aged T1DM mice. These findings might further guide recommendations related to the management of critically ill, older patients with T1DM admitted to ICUs and develop treatment guidelines addressing sex-specific differences. 

## 2. Materials and Methods

### 2.1. Animals 

All experiments were performed on 60 to 68 week old (n = 12) adult male and female heterozygous Ins2^Akita^ mice (C57BL/6-Ins2Akita/J; Strain# 003548), which develop type 1 diabetes (TD1, insulin-dependent) and were purchased from Jackson Laboratories (Bar Harbor, ME, USA). The size of the experimental group was calculated using an animal sample size calculator. All rodents were kept on a 12-h light/dark cycle during the experiment and were given unlimited access to food pellets and water. Experimental protocols were strictly followed and were approved by the Institutional Animal Care and Use Committee (IACUC#R011433) at the University of South Florida, Tampa, in compliance with the US National Institutes of Health (NIH).

### 2.2. Hyperoxia Exposure

The experimental groups were either subjected to normoxia (normal air) or hyperoxia (>90% O_2_) in an airtight chamber for 72 h. Both experimental groups had access to food and water ad libitum. Using an oxygen analyzer, the oxygen levels in an airtight container were regulated throughout the duration of the experiment (Vascular Technology, Chelmsford, MA, USA). After the completion of the experiment (72 h), the mice were put under isoflurane anesthesia for electrocardiography. Intraperitoneal injection (IP) of 50 mg/kg euthasol was used to euthanize the control and hyperoxia-exposed treatment groups in order to prepare for thoracotomy. The blood was immediately drawn and centrifuged at 12,500 rpm for 5 min for the collection of plasma serum. Each heart was dissected for the collection of atriums, septum, and right and left ventricles. The tissues were preserved at −80 °C for subsequent analysis. 

### 2.3. Physical Parameters

Body weight measurements of aged male and female Akita mice were taken before and after exposure to normoxia/hyperoxia. Changes in body weight were recorded in both sexes. All body weight measurements were normalized with the tibia length. As reported in our earlier article [14], lung wet-to-dry weight ratio was also measured in normoxic and hyperoxic groups to evaluate hyperoxia-induced lung edema. 

### 2.4. Histological Analysis

Hematoxylin and eosin (H&E) staining was performed on the excised hearts collected from aged Akita mice exposed to either normoxia or hyperoxia according to the previously published protocol [14]. Digital images of stained heart sections (10 µm thick) were taken using a Keyence BZ-X800 microscope, and the overall cross-sectional area was measured using ImageJ software. The overall cardiomyocyte surface area was measured using a fluorescently labeled wheat germ agglutinin (FITC-WGA, Sigma-Aldrich, Berkeley, MO, USA) and DAPI (Thermo Fisher Scientific, Waltham, MA, USA) stained heart section as described previously. Briefly, after fixation, sections were washed with phosphate-buffered saline (PBS) three times, and then stained with WGA (Sigma-Aldrich) for 25 min, followed by a second wash done three times in PBS. Sections were then mounted in a ProLong Gold antifade reagent with DAPI (Thermo Fisher Scientific) and imaged under a fluorescent microscope. ImageJ software was used to quantify cells in sections at 20× per group (n = 6). Relative cell area was calculated for each group by dividing each total section area per region counted by the number of cells within the counted region. The area of each cardiomyocyte in the cross-section was measured using ImageJ software for each group. The mean and standard deviations were represented in the form of a bar diagram.

### 2.5. Western Blotting

The experimental methods for protein quantification and analysis were all adapted from our previous publication [14]. Briefly, approximately 30 mg of left ventricle tissue was excised from both groups and homogenized in cell lysis buffer (Cell Signaling Technology, Danvers, MA, USA) containing a protease cocktail (Sigma Life Science, Burlington, MA, USA) and PMSF. The homogenized samples were centrifuged at 15,000 rpm for 30 min at 4 °C, and supernatant was quantified for 50 μg of equivalent proteins using the Pierce™ BCA Protein Assay Kit (Thermo Fisher Scientific). Each sample was denatured and loaded into an SDS-PAGE precast gel (Bio-Rad Laboratories, Hercules, CA, USA). Blots were later blocked in 5% *w*/*v* nonfat dry milk for 1 h and probed in 1:5000 dilutions of primary antibodies for Kv1.4 (AB5926) and GAPDH (Millipore) antibodies, and 1:1000 dilutions of KChIP2 (Abcam, Cambridge, MA, USA), Kv4.2 (Millipore, Billerica, MA, USA), estrogen receptor beta (Thermo Fisher, CA, USA), androgen receptor (Abcam, Cambridge, MA, USA), and Kv1.5 antibody (Santa Cruz Biotechnology, Dallas, TX, USA). The band intensities from GAPDH were used to normalize the target proteins using ImageJ software.

### 2.6. Statistical Analysis

All data sets were analyzed utilizing Student’s t-tests and a two-way ANOVA. Data analysis compared quantitative data populations of both normal distribution and equal variance, where a value of *p* ≤ 0.05 was considered statistically significant. Error bars are reported for all data groups as means ± SEM (standard error of the mean). 

## 3. Results

### 3.1. Physical Parameters

**Figure 1:**
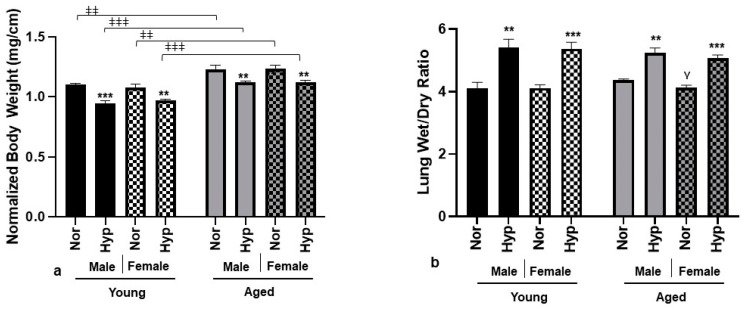

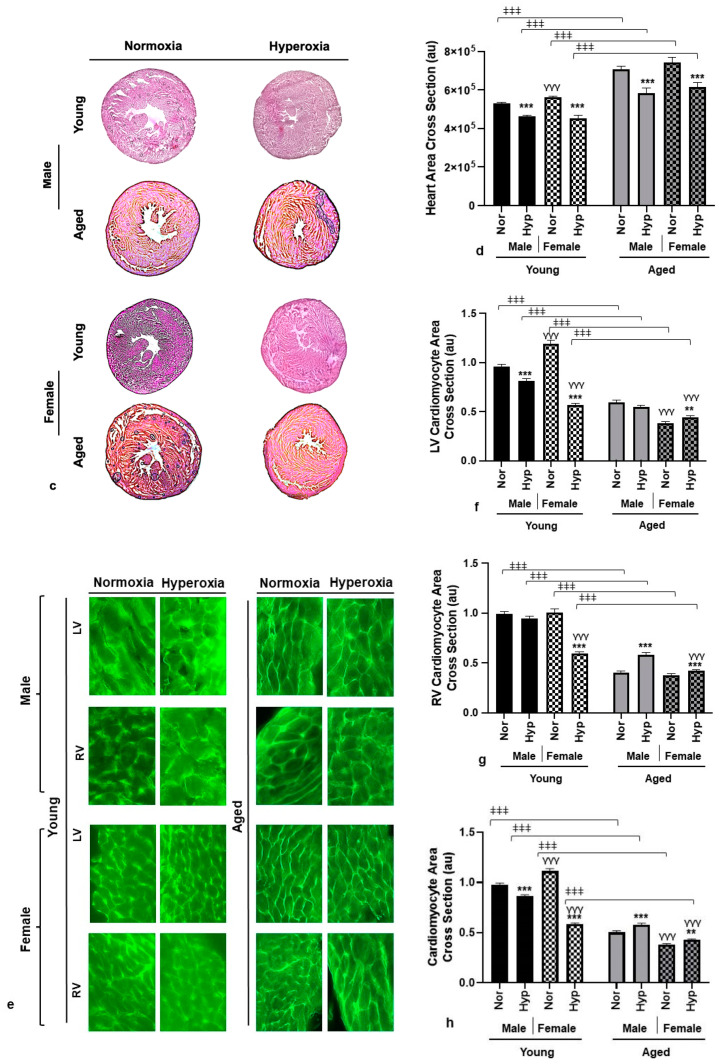
**Aged Akita mice showed significantly larger body weights and heart size, but smaller cardiomyocyte size compared to their young counterparts**. Body weight normalized to tibia length (g/cm) for all experimental groups (**a**), lung wet to dry weight ratio (**b**), H&E histological cross-sections (**c**), heart area cross-sections (au) from H&E stained hearts (**d**), WGA-stained cardiac myocytes from RV and LV (**e**), cardiac myocyte area of LV (**f**) and RV (**g**), and overall cardiomyocyte area pooled from LV, RV, and septum (**h**). All bar diagrams are mean (±SEM) of n = 6 per group. ** *p* < 0.005 *** *p* < 0.0005. * compares effects of hyperoxia and normoxia of same sex and age; Y compares male and female mice of the same age and treatment; ‡ compares young and aged mice from the same sex and treatment.

We performed a comparative analysis between young and aged male and female Akita mice before and after hyperoxia exposure (O_2_ > 90% for 72 h). The effect of hyperoxia on physical parameters like body weight and lung weight was analyzed. When compared to young mice, the body weight (normalized to tibia length) of aged mice was significantly higher regardless of the sex and exposure condition (Figure 1a). The hyperoxic groups significantly lost body weight irrespective of sex and age groups. No statistically significant sex-related body weight change was observed in young or aged groups. Hyperoxia exposure resulted in lung injury and manifested as lung edema. We assessed the lung wet to dry (W/D) ratio as an indication of successful hyperoxia exposure after 72 h. Both young and aged Akita mice showed a significant increase in lung W/D ratio without any sex-specific differences post-hyperoxia (Figure 1b).

### 3.2. Histological Analysis

As we observed a significant change in the normalized body weights after hyperoxia treatment in Akita mice, we also assessed heart size using H&E staining, and heart cross-sectional area was measured using ImageJ software. The normalized heart cross-sectional area of normoxia-treated aged Akita mice was significantly higher than their younger counterparts (Figure 1c,d). The results also showed that in Akita mice, exposure to hyperoxia decreased the heart cross-section irrespective of age and sex as compared to normal air controls. The histological modifications in the heart due to hyperoxia were also examined using WGA-stained cardiomyocytes (Figure 1e–h). The cardiomyocyte area was measured from the LV and RV of young and aged Akita mice (Figure 1f,g). A pooled average cardiomyocyte area (Figure 1h) was also plotted by pooling values from the RV and LV to investigate any differences in overall cardiomyocyte size between treatments, ages, and sexes. The pooled cardiomyocyte area for the aged group was significantly lower than the young group under normoxic exposure. Although we observed a significant decrease in the pooled cardiomyocyte area of young Akita mice after hyperoxia in both sexes, aged mice showed a significant increase in the pooled cardiomyocyte area irrespective of sex under hyperoxic conditions (Figure 1h). Another significant change observed is that while young female mice showed an increase in cardiomyocyte area as compared to the young males at normal air, aged female mice showed a significantly smaller cardiomyocyte area compared to their male counterparts at normal air (Figure 1h).

### 3.3. Electrophysiological Parameters

**Figure 2:**
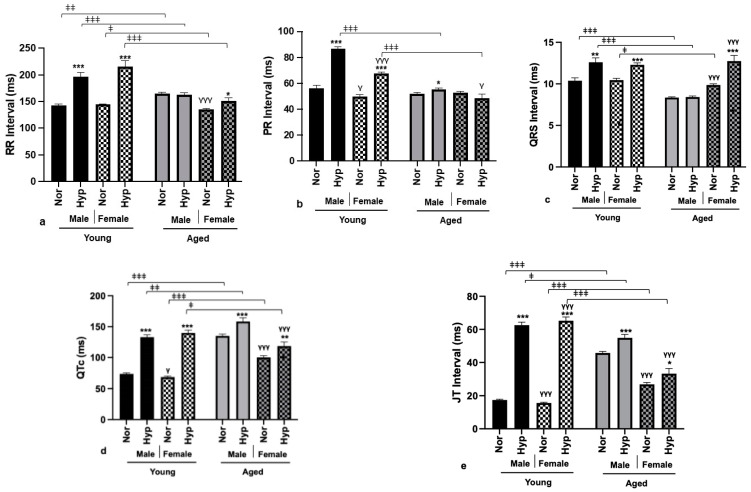
**Pre-existing electrical disparities in aged Akita mice.** ECG analysis in lead II mode in normoxia/hyperoxia treated aged and young male and female Akita mice. RR interval (**a**), PR interval (**b**), QRS interval (**c**), QTc interval (**d**), and JT interval (**e**). All bar diagrams show mean (±SEM) of n = 12 per group. * *p* < 0.05, ** *p* < 0.005, *** *p* < 0.0005. * compares effects of hyperoxia and normoxia of same sex and age; Y compares male and female mice of the same age and treatment; ‡ compares young and aged mice from the same sex and treatment.

As we observed significant changes in the physical parameters of aged Akita mice due to hyperoxia treatment, we also assessed changes in electrophysiological parameters by recording surface electrocardiograms (ECGs) in lead II mode in all groups. The QTc and JT intervals in the aged Akita group, regardless of sex, were prolonged under normoxia compared to the young group (Figure 2d,e). Nevertheless, under normoxic exposure, the QRS interval did shorten for both sexes (Figure 2c). Further, focusing on the changes only in the aged mice, we observed sex-specific differences under normoxic conditions, as prolonged RR, QTc, and JT intervals were seen in male mice compared to female mice. When compared between normoxic and hyperoxic groups, a significant increase in QTc and JT intervals was seen in both aged males and females after hyperoxia. However, RR and QRS intervals were significantly increased for aged females under hyperoxia (Figure 2a,c), with no significant change in the PR interval. In contrast, aged males showed a significant increase in the PR interval after hyperoxia, while the females exhibited no change (Figure 2b). 

### 3.4. Gene Expression

**Figure 3:**
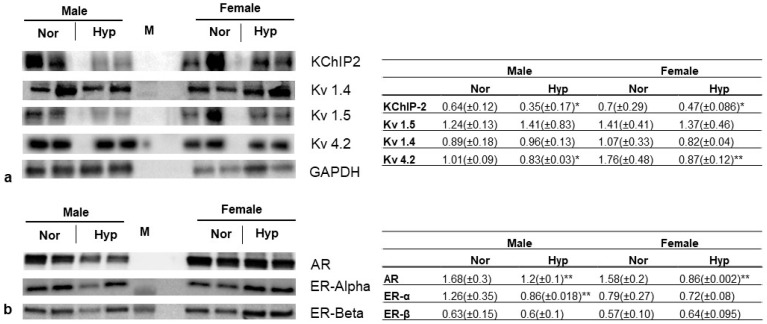
**Protein profiles of key potassium channels and sex hormone receptors in hearts of aged Akita mice.** Key potassium channels and their auxiliary proteins such as KChIP2, Kv1.4, Kv1.5, and Kv4.2 and housekeeping gene GAPDH (**a**), and androgen receptor (AR) and estrogen receptors (ER-α and β) (**b**). Values shown in the tables are mean (±SE) normalized band intensities from n = 4 per group. * represents *p* < 0.05 and ** *p* < 0.005 between normoxia and hyperoxia.

The observed changes in the electrical parameters of aged Akita mice suggest changes in the molecular expression of key cardiac ion channels. In this research, we have focused on analyzing key potassium ion channel genes along with their auxiliary binding protein from the left ventricle. The selected potassium ion channels are Kv 4.2, Kv 1.4, Kv 1.5, and KChIP2, as they are sensitive to oxygen levels. Our data showed that exposure to hyperoxia decreased the protein levels of potassium ion channels like Kv 4.2 and KChIP2 in both male and female aged Akita mice (Figure 3a). However, we did not observe any significant changes in the protein levels of Kv 1.4 and Kv 1.5 in hyperoxia-treated aged mice, both male and female (Figure 3a). We then analyzed androgen receptor (AR) and estrogen receptor (ERα and ERβ) expression in aged mice using Western blotting. Our data showed a significant decrease in AR in both male and female aged mice compared to their normoxia controls (Figure 3b), whereas a significant decrease in ERα level after hyperoxia was observed only in aged males, with no significant change in their female counterparts. No significant change in ERβ levels was observed in both male and female aged Akita mice after hyperoxia treatment. 

## 4. Discussion

Long-term hyperoxia has been associated with unfavorable outcomes, including severe cardiac remodeling, thereby increasing the risk of developing CVD. This investigation expands upon two prior rodent studies performed by our group [14,21]. In the first investigation, we found that even in normoxic conditions, mice with T1D had diminished cardiac function compared to WT mice. Additionally, hyperoxic exposure further increased the cardiac risks, resulting in abnormal ventricular depolarization [21]. The results of the second study showed that aging was another distinct risk factor for cardiac remodeling, and exposure to hyperoxia exacerbated the cardiac physiological and functional characteristics [14]. As a result, the focus of this study was to examine whether hyperoxic exposure in the presence of two detrimental variables like advanced age and preexisting diabetic complications (T1D) would cause a stepwise increase in the risk of developing CVD. In both prior investigations, we observed distinct sex-related differences based on the risk variables. In young T1D mice, for example, males had a higher chance of developing the worst cardiac outcomes than females [21]. As a result, this research also aims to highlight sex-related heterogeneity in responses between aged T1D male and female mice. The main emphasis of this work is to investigate the impact of hyperoxia on cardiac function in a murine model with advanced aging and developed T1DM. To examine the effect of age as a cardiac risk factor, we performed group comparisons using our previously published work in young Akita mice (T1D) as a reference. As this research studies the effects of comorbid conditions like type 1 diabetes, hyperoxia, and advanced age, the results of this research can assist in refining the ICU protocols for T1D patients of advanced age who undergo MV in ICU units.

Consistent with our earlier findings [14,23], exposure to hyperoxia for 72 h resulted in approximately 10–20% of body weight loss in Akita mice, irrespective of sex and age (Figure 1a). Barazzone et al. [24] reported similar hyperoxia-induced body weight loss in WT mice as a result of a six-fold increase in serum leptin levels, a cytokine regulating food intake. However, the specific reason for weight loss in hyperoxic aged Akita mice remains to be investigated. Clinically, in MV patients, depending upon the length of the ICU stay, weight loss is commonly observed due to underfeeding, muscle wasting, and loss of lean tissue mass [25]. When comparing between two age groups, aged Akita mice showed an increase in body weight normalized to tibia length as compared to the young Akita mice, irrespective of exposure condition and sex (Figure 1a). This baseline obesity in aged Akita mice is consistent with clinical observations and could be attributed to two factors: advanced age and/or the presence of type 1 diabetes (T1D). The widespread consensus in humans is that body weight increases linearly with age [26], due to the accumulation of fat mass, physical inactivity, a declining basal metabolic rate, or hormonal changes [27]. On the other hand, despite the fact that traditionally type 1 diabetic patients have been thought to have lower body weights, the latest clinical studies report otherwise, stating that 50% of T1D patients are either overweight or obese [28]. However, the weight gain in T1D patients may not be related to age but rather due to clinical factors like insulin therapy [29]. This increase in body weight of older T1D patients is linked to the increased risk of CVD complications [30,31]. Although in the Akita model we did not find sex differences in body weight increase, Szadkowska et al. [28] reported that young diabetic females had a greater amount of fat mass than diabetic males. Kawamura et al. [32] showed that long-term exposure (60 h) to hyperoxia in rats can result in the generation of ROS, which are capable of targeting vulnerable alveolar epithelial and alveolar capillary endothelial cells. This can progress to fluid accumulation, edema, and irreversible lung injury. The lung wet-to-dry (W/D) ratio is an index of the lung water content, and a reading above 4 usually indicates pulmonary edema [33]. In the present study, 72 h of hyperoxia exposure in Akita mice of both sexes and age groups resulted in an increase in the lung W/D ratio above 4, serving as an indicator of successful hyperoxic exposure and lung injury (Figure 1b).

The cross-sectional area of the heart was measured as an indicator of cardiac hypertrophy. Cardiac hypertrophy is an independent predictor of CVD; however, a previous report from our lab showed that the presence of hyperoxia-induced cardiac hypertrophy in young male WT mice is an adaptive response that may be beneficial to maintain cardiac contractile function [34]. Interestingly, this protective cardiac hypertrophy was absent in aged male WT mice [14]. In this study, we observed that regardless of age groups, male Akita mice did not show any signs of compensatory cardiac hypertrophy to counter hyperoxic stress (Figure 1c,d). The reproductive hormone testosterone is pro-hypertrophic and binds to the androgen receptors (ARs) expressed in cardiac myocytes [35]. Post-binding, testosterone induces cardiac hypertrophy by both increasing glucose uptake by AMP-activated protein kinase (AMPK) and activating the rapamycin complex 1 (mTORC1) pathway [36]. Moreover, ARs permit regulation of cardiac hypertrophy in the presence of testosterone. The absence of compensatory hypertrophy in hyperoxic aged Akita mice could be explained at least in part by the decreased expression of cardiac ARs (Figure 3b). 

Normoxia-treated aged Akita female hearts were of comparable size to the aged male hearts (Figure 1d). However, this contrasts with the previously reported observation in normoxia-treated aged WT mice, where female mice had significantly smaller heart size than male mice [14]. We also observed an age-related heart size difference between young and aged Akita mice. Aged hearts normalized to tibia length were significantly larger than young hearts in both sexes, irrespective of exposure conditions. However, this increase in heart size was not due to advanced age but was related to the effects of T1D. We interpret this based on our previous report, as aged WT mice did not demonstrate an increase in heart size when compared to young WT mice [14]. 

Another age-related difference in Akita mice was the overall decrease in cardiomyocyte area in aged compared to young individuals (Figure 1e,h). This is consistent with a previous report on aged WT mice, where cardiomyocyte shrinkage as a result of progressive cardiomyocyte apoptosis was due to the effect of advanced age alone [14]. This decrease in cardiomyocyte size in aged Akita mice, despite their larger heart size (Figure 1c,d), is plausibly either due to interstitial edema, which is commonly observed after coronary occlusion as a response to cardiac injury, specifically in diabetic patients [37], or due to diabetes-induced cardiac fibrosis [38], which needs to be further investigated. We also observed a significant increase in cardiomyocyte area in aged male and female hearts after hyperoxia treatment (Figure 1e,h) despite reduced heart size under hyperoxia (Figure 1c,d), which may be due to transient homotypic fusion of cardiomyocytes resulting in large, multinucleated cardiomyocytes [39,40,41] as a response to hyperoxia-induced cardiac injury.

Exposure to high levels of oxygen corresponds to the increased susceptibility of developing cardiac arrhythmias [42]. Thus, we measured common ECG parameters like RR, PR, QRS, QTc, and JT intervals post hyperoxia as an independent predictor of future risk of CVD. In young Akita mice of both sexes, exposure to hyperoxia resulted in increased RR, PR, QRS, QTc, and JT intervals (Figure 2a–e), whereas in aged males and females, hyperoxia prolonged the QTc and JT intervals (Figure 2d,e). Prolonged QTc and JT intervals indicate abnormal repolarization and intraventricular conduction delay, predisposing the aged diabetic population to the early development of ventricular arrhythmias and acute myocardial infarction [43,44]. Importantly, in diabetic patients, prolongation of the QT interval is a quantifiable measure of risk for CVD [45]. The QT interval represents the total duration of depolarization (QRS complex) and repolarization (T wave) of ventricular myocytes. As per clinical observations, a prolonged heart-rate-corrected QT interval (QTc interval) was a long-term predictor of mortality in patients with T1DM [46]. Moreover, on comparing between age groups, aged Akita mice of both sexes had prolonged QTc and JT intervals even when at normal air (Figure 2d,e). This indicates that advanced age would represent a preexisting risk factor for T1DM patients, and exposure to hyperoxia would further exacerbate the condition. A peculiar sex-based difference in both young and aged Akita mice was the presence of prolonged QTc in normoxia-treated males rather than the commonly observed phenomenon in females [23]. Additionally, we observed that male Akita mice in the aged group showed a significantly higher resting heart rate than age-matched females (Figure 2a). Therefore, aged male Akita mice may be at higher risk of facing adverse outcomes under normal air due to the worsening of the QTc interval, a high resting heart rate, and the absence of protective cardiac hypertrophy. Taken together, observations in aged Akita mice suggest that T1DM patients of advanced age, especially men, have higher chances of experiencing negative consequences, like developing intraventricular arrhythmias, after hyperoxia exposure. 

Another interesting observation in aged Akita mice is significantly lower PR intervals after hyperoxia compared to their young counterparts, irrespective of sex (Figure 2b). The PR interval measures the time taken for an electrical impulse to be generated in the sinus node and the advancement of that impulse through the atria towards the atrioventricular (AV) node in the ventricles. As a result, a prolonged PR interval indicates a higher risk of developing atrial fibrillation [47] and is linked to an increased risk of heart failure and mortality [48]. Thus, young T1DM patients would have higher odds of developing atrioventricular conduction abnormalities as compared to older patients.

The prolonged action potentials are caused by the dysfunction of ion channels like sodium, potassium, and calcium channels. The inward depolarizing (Na^+^ and Ca^2+^) and outward repolarizing (K+) currents are a result of the synchronous opening and closing of cardiac ion channels. Ion channel remodeling can also occur during cardiac hypertrophy and failure [49]. We have previously shown that hyperoxia results in dysregulation of cardiac potassium channels in wild-type aged and young rodents of both sexes [14,34]. In this study, to analyze whether the conduction abnormalities after hyperoxia arise from the expression changes in the cardiac K^+^ channels, we performed Western blot analysis on Kv 1.4, Kv 4.2, Kv 1.5, and KChIP2. Although we did not observe any significant changes in Kv 1.5 and Kv 1.4 in aged Akita mice, a significant decrease in Kv 4.2 and KChIP2 was observed in aged male and female Akita mice (Figure 3a). Barry et al. [50] showed that a point mutation in Kv 4.2 results in functional knockout of transient outward potassium currents (*I_to_*), resulting in a prolonged QT interval. This also demonstrates that cardiac electrical modeling can result from altered expression of endogenous K^+^ channels. The post translational levels of an auxiliary subunit of potassium channel interacting protein 2 (KChIP2) are decreased in the absence of the Kv4.2 subunit. Consequently, our data showed a parallel decrease in levels of KChIP2 in both male and female aged Akita mice. Functional reduction of *I_to_* currents due to a reduction in KChIP2 contribute to the progression of heart failure [51]. KChIP2 mRNA and protein were reported to be significantly downregulated in human heart failure [52]. These data show that the presence of T1DM adds another layer of complexity to the electrophysiological remodeling seen in heart failure, specifically under hyperoxia. 

## 5. Conclusions

MV is an essential part of supportive care in ICUs, but it is not without risk to physiological and cardiovascular function [53,54]. One of the major drawbacks of oxygen therapy is exposure to hyperoxia, which is an independent factor increasing the risk of the onset of CVD [23]. The severity of cardiac outcomes depends on factors like age, sex, and preexisting comorbidities. In this report, we studied the combination of two vulnerability elements: advanced age and T1D complication. Aged Akita mice face innate cardiac disadvantages as compared to young Akita mice. Thus, in aged Akita mice, we observed body weight gain, increased heart cross-sectional area, prolongation of QTc and JT intervals, and dysregulation of KChIP-2 and Kv 4.2 cardiac channels even under normal air. As compared to male WT mice, male Akita mice did not show protective compensatory hypertrophy against hyperoxic stress. These observations indicate the presence of innate challenges due to advanced age and T1D. Further exposure to hyperoxia resulted in decreased body weight and heart size, signs of lung edema, major prolongation of the QRS, QTc, and JT intervals, and reduction in cardiac potassium channels in aged Akita mice. We also demonstrated sex-specific differences in an aged Akita murine model, as the baseline RR, QTc, and JT intervals were significantly higher in males than in females. These findings help identify the increased cardiovascular risk in aged Akita mice when exposed to hyperoxia and would guide regulation of supplemental oxygen therapy for aged patients admitted to ICUs with preexisting conditions like T1DM.

### Limitations and Challenges

Although mice are a commonly used animal model in cardiovascular research, their high heart rates, small size, short lifespan, and differences in both cardiac ion channel expression and contractile function necessitate an animal model that better reflects parameters observed in human patients, as this will heighten the translational value and allow us to investigate these changes accurately.

Rearing and maintaining Akita (C57BL/6-Ins2Akita/J; Strain #:003548) mice was one of the biggest challenges in this project, as these mice have an average lifespan of 305 days, and very few mice reached 60 to 68 weeks of age that were suitable for our study. Jackson laboratories sell Akita mice of maximum 4 to 5 weeks of age, so we had to wait for more than a year to get the desired age for this study. Many mice were eliminated from the study due to age-related mortality in addition to hyperoxia-induced deaths. Therefore, we used Akita mice very conservatively for histological and molecular (Western blotting) experiments.

## Data Availability

All data obtained in this research will be available from MDPI upon request.
